# Associations between toddlers’ and parents’ BMI, in relation to family socio-demography: a cross-sectional study

**DOI:** 10.1186/s12889-015-2602-8

**Published:** 2015-12-17

**Authors:** Marie Lindkvist, Anneli Ivarsson, Sven Arne Silfverdal, Eva Eurenius

**Affiliations:** Department of Public Health and Clinical Medicine, Epidemiology and Global Health, Umeå University, SE 901 87 Umeå, Sweden; Department of Statistics, USBE, Umeå University, SE 901 87 Umeå, Sweden; Department of Clinical Science, Paediatrics, Umeå University, SE 901 87 Umeå, Sweden

**Keywords:** Body mass index, Children, Cross-sectional studies, Family characteristics, Family health, Obesity, Overweight, Socioeconomic factors

## Abstract

**Background:**

It is well established that the pregnancy and the first years of life are important for future childhood health and body weight. Even though current evidence suggests that both parents are important for childhood health, the influence that parents’ BMI and socio-demography has on toddlers’ BMI has so far received little attention. This study aimed to increase our knowledge on the association between toddlers’ and parents’ BMI, in relation to family socio-demography. Further, the aim was to investigate the interaction between the mothers’ and fathers’ BMI in relation to their child’s BMI.

**Methods:**

A total of 697 children with a median age of 18 months (range 16-24 months) participated in the study along with their mothers (*n* = 697) and fathers (*n* = 674). As regards representability, our parental sample had a lower proportion of immigrants and the parents were more gainfully employed compared to parents in the rest of Sweden (when the child was 18 months old). The parents completed a questionnaire on parental and child health. Data on parental weight, height, and socio-demographics were recorded along with the child’s weight and height measured at an ordinary child health care visit. We used the thresholds for children’s BMI that were recommended for surveillance by the Royal College of Paediatrics and Child Health in 2012 based on the WHO reference population.

**Results:**

Among the toddlers, 33 % had a BMI above the WHO 85^th^ percentile and 14 % had a BMI above the WHO 95^th^ percentile. The probability of a toddler having a BMI above the WHO 95^th^ percentile was significantly increased if either the mother or father was overweight (BMI ≥ 25 kg/m^2^). Furthermore, we found a positive synergistic effect between the mother and father being overweight and their child having a BMI above the WHO 85^th^ percentile. No associations were found between the toddlers’ BMI and the family’s socio-demographics, but there were associations between the parents’ BMI and the family’s socio-demographics.

**Conclusion:**

High BMI is common even in toddlers in this population. The risk increases if one parent is overweight, and it increases even more if both parents are overweight. The results in this study confirm the importance of considering familial risk factors when examining child health and BMI at ordinary child health care visits already at an early age.

## Background

According to the World Health Organization, overweight and obesity are the most important public health threats and are now associated with more deaths worldwide than underweight [1]. The obesity epidemic varies between countries, and in high income countries obesity affects both sexes and all ages but is more prominent in the most disadvantaged groups [2]. Overweight/obesity is common among expectant parents, and previous studies have found overweight/obesity for about 53 % of men and for about 30 % of women among expectant parents in Sweden [3, 4]. A clear socioeconomic association was found with higher odds of being obese the lower the individual’s social status level, and BMI seems to be correlated within couples [3]. The prevalence of obesity has also increased among children over the last three decades [5], and about 15 % of 4-year-old children in Sweden are overweight and 3–4 % are obese [6]. A recent systematic review showed that maternal pre-pregnancy overweight was associated with childhood overweight at follow-up at a median age of 6 years (range 2–14 years) [7]. It is well established that infancy weight and body composition up to 4 years of age are important predictors for childhood obesity [8–11]. Children with excessive weight, and especially those with a rapid weight gain early in life, are at risk of adult obesity [12, 13] and unfavourable health outcomes, especially if their parents are obese [14]. It has also been found that BMI at 4 years of age is related to paternal BMI [15]. Current evidence suggests that both parents are important for childhood health, and parents with poor self-rated health are more likely to have children with child health disorders during the first three years of life [16, 17]. However, analysis of childhood obesity prevalence and investigation of the potential causal associations are required in order to implement and assess the effectiveness of different interventions [18].

A BMI ≥ 85^th^ percentile at 2 years of age and at preschool age in the US seems to be a powerful predictor of kindergarten overweight [19]. However, no data for Swedish toddlers aged 1–2 years are yet available, and the influences of parental BMI and socio-demography on toddlers’ BMI already at this early age have so far received little attention. The aim of this study was to increase our knowledge on the association between toddlers’ and parents’ BMI, in relation to family socio-demography. Further, the aim was to investigate the interaction between the mothers’ and fathers’ BMI in relation to their child’s BMI.

## Method

### Study context

The study was conducted in Västerbotten County in Sweden, which has 260,000 residents and about 3,000 births annually. In Sweden, Child Health Care (CHC) is free of charge and reaches nearly all children aged 0–6 years. The county has about 40 CHC centres staffed with child health nurses and family doctors. Each child visits the CHC centre about 11 times during the first 1½ years of his/her life and then at 3, 4, and 5½ years of age.

Local data revealing an alarming prevalence of overweight and obesity among 4 year olds [20] resulted in the County Council launching the Salut Child-Health Intervention Programme [4, 21, 22]. The countywide programme consists of age-adapted interventions starting with the parents-to-be and continuing to follow the child up to 18 years of age. The programme includes epidemiological surveillance.

### Study design and data collection

A cross-sectional study design was used. Because the questionnaire was only in Swedish, those parents who could not read Swedish were excluded. The questionnaire was implemented gradually in the health care region to test and implement the questionnaire within ordinary health care because research was not the main priority when the data collection started. Data collection was carried out in those seven CHC centres that succeeded in implementing the questionnaire. Once the questionnaire was introduced all parents belonging to one of these CHC centres received a letter with information and a questionnaire, and a request to bring it completed to the CHC centre at their child’s ordinary health and development check-up at approximately 1½ years of age. From the start, all written communication and questionnaires contained the information normally required by an ethical board when research is planned. Among other things, participants were informed that their returned questionnaires might in the future be used in research approved by an ethical board and that they could choose not to fill in the questionnaire without any consequences for the health care they would receive. The questionnaire covered child and parental health and living conditions, and height and weight data were added at the visit. Child health nurses at seven CHC centres were involved in the data collection from April 2008 to June 2012. A total of 697 children aged between 16 and 24 months participated in the study along with their mothers (*n* = 697) and fathers (*n* = 674).

### Questionnaire – items and definitions

The parental part of the questionnaire was a shortened version of a questionnaire used within antenatal health care since 2008 [4]. Questions on parental self-reported weight, height, and socio-demographics were duplicated so that both parents answered the same questions separately. The child part of the questionnaire contained parent reports about the child’s health and living conditions. The items included in this study are described below.

#### Socio-demography

*Maternal* and *paternal age (years)* and *child age (months and days)* were calculated using the date for the check-up visit for the child at the CHC centre and the birth dates of the parents and the child.

*Parental educational level* was categorized as ≤9 years, 11–12 years, >12 years, or university degree based on the question “What is the highest level of education that you have completed?” with the following eligible five answers: 1) Less than 9 years of schooling, 2) Compulsory school or the equivalent of 9 years of schooling, 3) Secondary school or the equivalent of 12 years of schooling, 4) Post-secondary education, less than three years, and 5) Post-secondary education, three years or more.

*Employment status* was categorized as employed (including self-employed), student, unemployed, on parental leave, or other (i.e. homemakers, on sick leave, or retired) based on the question “What is your present type of occupation?” with the following eligible eight answers: 1) Employed, 2) Student, apprentice, 3) Self-employed, 4) Doing household work at home (no personal income), 5) Jobseeker for more than 6 months, 6) On parental or other leave, 7) Jobseeker for less than 6 months, and 8) On sickness, old age, or disability benefit.

*Family situation* was categorized as the child living with either both parents, alternating between the mother and father, or living with a single mother. Having or not having any siblings/half-siblings was based on the question “With whom is the child living? (tick one or several boxes)” with the following eligible answers: Its mother, Its father, Its siblings/half-siblings, Its stepmother (Its father’s new wife/partner), Its stepfather, Alternating between its mother and father, and Other.

*Type of housing* was categorized into villa or townhouse area, house outside urban areas, or apartment based on these three eligible answer alternatives to the question “What type of housing does your family have?”

*Day care* was categorized as municipal childcare, at home with the mother, or at home with the father.

*Parental country of origin* was categorized as Sweden or other countries.

#### BMI for toddlers

The *weight* and *length* of the toddler were measured by the child health nurse at the CHC centre. Children’s BMI changes considerably between birth and adulthood, and the thresholds that are used must take into account the child’s age and sex. For population monitoring in this age group, the 2^nd^ , 85^th^, and 95^th^ percentiles of the WHO Child Growth standard are often used [23, 24], and in this study these are referred to as WHO 2^nd^ percentile, WHO 85^th^ percentile, and WHO 95^th^ percentile.

#### Parental BMI

*BMI* was calculated by dividing self-reported body weight (kg) by height (m^2^) and categorized for the parents in accordance with the WHO standard [25] as *underweight*: ≤18.49, *normal weight*: 18.50–24.99, *overweight*: 25.0–29.99, and *obesity*: ≥30.0 kg/m^2^. Data for pregnant mothers were excluded in the analysis of BMI.

### Statistical analysis

Most analyses were performed using SPSS version 21. Descriptive results are presented as medians, ranges, numbers, and percentages. Characteristics of mothers/girls and fathers/boys were compared using the chi-square test for categorical data and the Mann–Whitney test for quantitative data. Thresholds for children’s BMI were calculated making use of LMS Growth version 2.77 [26], which uses each child’s age in days to obtain as exact a threshold as possible. Crude and adjusted logistic regression analyses were used for investigating the associations between the toddlers’ BMI group (the dependent variable) and the parent’s BMI group and socio-demographic variables (the independent variables). In the adjusted analyses between toddler BMI and parental BMI, socio-demographic variables with *p*-values < 0.10 in the crude analyses were included. Results from the logistic regression analyses are presented as odds ratios (OR) with corresponding 95 % confidence intervals. Relative excess risk due to interaction (RERI) was calculated for investigating additive interactions [27] between the mother’s and father’s reported overweight/obesity in relation to their child’s BMI status. Positive departure from 0 of RERI indicates an additive interaction. Statistical significance was defined as *p* < 0.05.

### Comparison of the sample with national data

We used data and selected variables from Statistics Sweden—as accessed through the Umeå SIMSAM Lab (www.org.umu.se/simsam/english/) – in order to check the representativeness of our study population. We compared certain characteristics (i.e. post-secondary education, gainful employment, and country of origin) of the parents of all children in the country that turned 1½ years old during 2008–2012 with the parents in our study population.

### Ethical considerations

Informed consent was given by the parents, and reporting was limited to the group level. Ethical approval was obtained from the regional ethical review board in Umeå for the Salut Programme (Dnr 2010-63-31 M) and for the Umeå SIMSAM Lab (Dnr 2010-157-31Ö).

## Results

### Characteristics of the study participants

The median age of the toddlers was 18 months (range 16–24 months), the median age for the mothers was 31 years (range 19–48 years), and the median age for the fathers was 33 years (range 19–59 years) (Tables 1 and 2). Almost all of the toddlers lived with both parents (96 %), and half of them had siblings (Table 1). The majority of the families (59 %) lived in a villa or townhouse, 26 % lived in houses outside urban areas, and 15 % lived in apartments. The majority of the toddlers had municipal childcare (57 %), one third stayed at home with the mother, and the rest stayed at home with the father. The mothers had a higher educational level, while the fathers were to a greater extent employed. Only a few of the parents were born outside Sweden (Table 1).

**Table 1:** Socio-demographic characteristics of families in the study

Socio-demographic characteristics	Mothers	Fathers	*p*-value	Families	
	*n* = 697	*n* = 674		*n* = 697	
Educational level, *n* (%)			<0.001^1^	Family formation, *n* (%)	
≤9 years	41 (6)	21 (3)		Both parents	663 (96)
10–12 years	206 (30)	314 (50)		Alternating between mother and father	9 (1)
>12 years	118 (17)	88 (14)		Single mother	20 (3)
University degree	320 (47)	209 (33)		Siblings/half-siblings, *n *(%)	
Employment status, *n *(%)			<0.001^1^	Yes	355 (51)
				No	342 (49)
Employed	439 (66)	552 (86)		Type of housing, *n* (%)	
Parental leave	104 (16)	45 (7)		Villa or townhouse	404 (59)
Student	47 (7)	19 (3)		House outside urban areas	181 (26)
Unemployed	58 (9)	22 (3)		Apartment	101 (15)
Other	19 (3)	7 (1)		Day care, *n* (%)	
Country of origin, n (%)			0.086^1^	Municipal childcare	360 (57)
Sweden	649 (93)	639 (95)		At home with mother	202 (32)
Other	46 (7)	30 (5)		At home with father	68 (11)
Age, median (range)	31 (19–48)	34 (19–59)	<0.001^2^		

^1^Mothers and fathers compared using the chi-square test

^2^Mothers and fathers compared using the Mann–Whitney test

**Table 2:** BMI and age of children in the study

	Total	Girls	Boys	*p*-value
	*n* = 697	*n* = 355	*n* = 342	
		(51 %)	(49 %)	
BMI^1^, n (%)				
<2^nd^	3 (0.5)	2 (0.6)	1 (0.3)	0.542^2,3^
2^nd^ – 84^th^	421 (66.0)	212 (64.4)	209 (67.6)	
85^th^ – 94^th^	125 (19.6)	70 (21.3)	55 (17.8)	
95^th^ – 100^th^	89 (13.9)	45 (13.7)	44 (14.2)	
Age (months), median (range)	18 (16-24)	18 (16-24)	18 (16-24)	0.132^4^

^1^Weight and height were measured at the child health care centre and were used for calculating BMI in kg/m^2^

^2^Girls and boys were compared using the chi-square test

^3^For calculation of the *p*-value, the first two BMI categories were merged because of the low numbers of toddlers in the category < 2^nd^

^4^ Girls and boys were compared using the Mann–Whitney test

### Comparison of the sample with national data

Comparison of our sample with national population data showed that parental post-secondary education was similar in both samples (mothers 53 % and 50 %, fathers 39 % and 40 %, respectively). However, gainful employment was higher among our parents compared to all parents in the country (mothers 82 % and 72 %, fathers 95 % and 87 %, respectively). The largest difference was found concerning country of origin as 95 % of our mothers were from Sweden compared to 78 % among the mothers from the whole country (data not shown).

### BMI for toddlers

BMI was above the 85^th^ percentile for 33.5 % of the toddlers in our sample and above the 95^th^ percentile for 13.9 % according to the WHO reference values (Table 2). BMI below the WHO 2^nd^ percentile was unusual (0.5 % of toddlers in our sample). No significant differences were found between girls and boys in either BMI status or age (Table 2).

### Parental BMI

The median BMI for mothers was 23.2 kg/m^2^. and the median BMI for the fathers was 25.4 kg/m^2^. (Table 3). Being overweight or obese was common, and there was a statistically significant difference between the sexes for weight status with more fathers being overweight or obese compared to mothers (54 % vs. 31 % accordingly, *p* < 0.001) (Table 3). There was a significant within-couple association concerning BMI group, and the odds of being overweight or obese increased significantly relative to the partner’s overweight/obesity (OR = 1.78, CI 1.23–2.58). The odds of being obese if the partner was obese was even higher – OR = 3.92 (CI 2.00–7.66) – compared to if the partner was not obese.

**Table 3:** BMI of parents in the study

	Mothers	Fathers	*p*-value^3^
	*n* = 632^1^	*n* = 674	
BMI^2^, *n* (%)			<0.001
Underweight	22 (4)	3 (1)	
Normal weight	381 (65)	298 (45)	
Overweight	125 (21)	280 (43)	
Obesity	55 (10)	72 (11)	
BMI^2^, median (range)	23.2 (16.2–48.4)	25.4 (18.4–54.9)	

^1^Due to pregnancy, 65 mothers were excluded

^2^Self-reported weight and height were used

^3^Mothers and fathers were compared by using the chi-square test

### Association between parental BMI and socio-demographics

There were significant associations between parental BMI and socio-demographics (data not shown). The mother’s obesity was significantly associated with low education and no employment for mothers and with low education for fathers. Furthermore, mothers’ overweight/obesity was significantly associated with low education for fathers, and fathers’ overweight/obesity was significantly associated with no employment for mothers.

### Associations between the toddler’s BMI and parents’ BMI

Further analysis was performed dividing the families into the following four groups depending on the BMI category for the parents: neither was overweight/obese, only the mother was overweight/obese, only the father was overweight/obese, or both were overweight/obese. Families where neither of the parents were overweight/obese were 34 % of the total, and families where both of the parents were overweight/obese were 20 % of the total (Table 4). Constructing one variable from those four groups – where the group “neither was overweight/obese” served as the reference – made it possible to investigate additive interactions between mother’s and father’s overweight/obesity on their child’s BMI status.

**Table 4:** Associations between parental and child BMI

Parental BMI (within couples)		Child BMI above the reference 85^th^ percentile			Child BMI above the reference 95^th^ percentile		
	*n* (%)	*n* (%)	OR (CI)	OR (CI)	*n* (%)	OR (CI)	OR (CI)
	*n* = 553		Crude^1^	Adjusted^1,2^		Crude^1^	Adjusted^1,3^
Neither overweight/obese	191 (30)	51 (28)	1	1	15 (8)	1	1
Only mother overweight/obese	61 (10)	21 (32)	1.20 (0.62–2.31)	1.26 (0.65–2.47)	11 (17)	2.68 (1.10–6.53)	2.66 (1.09–6.52)
Only father overweight/obese	192 (30)	59 (31)	1.14 (0.72–1.79)	1.11 (0.69–1.78)	32 (17)	2.40 (1.21–4.77)	2.31 (1.15–4.65)
Both overweight/obese	109 (17)	58 (47)	2.47 (1.48–4.11)	2.48 (1.47–4.21)	22 (18)	2.95 (1.40–6.22)	2.85 (1.34–6.06)
RERI			1.13	1.11		−1.13	−1.12

^1^Crude and adjusted logistic regression was used for the calculation of OR. In the adjusted analyses, socio–demographic variables with *p*–values <0.10 in the crude analyses were included

^2^Analysis adjusted for mothers’ employment, siblings, and mothers’ country of origin

^3^Analysis adjusted for mothers’ employment and siblings

The prevalence of toddlers being above the WHO 85^th^ percentile and having no parent overweight/obese was 28 %, and this prevalence was 47 % if both parents were overweight/obese (Table 4). Crude logistic regression analyses showed that the odds for children having a BMI above the WHO 85^th^ percentile were significantly higher if both the mother and the father were overweight/obese (OR 2.47, CI 1.48–4.11) compared to those who had no parent overweight/obese. Regarding the WHO 95^th^ percentile, children in families where at least one of the parents were overweight/obese had significantly higher odds (OR = 2.40, OR = 2.68, and OR = 2.95) of being above the threshold compared to those who had no parent overweight/obese (Table 4).

**Fig. 1:**
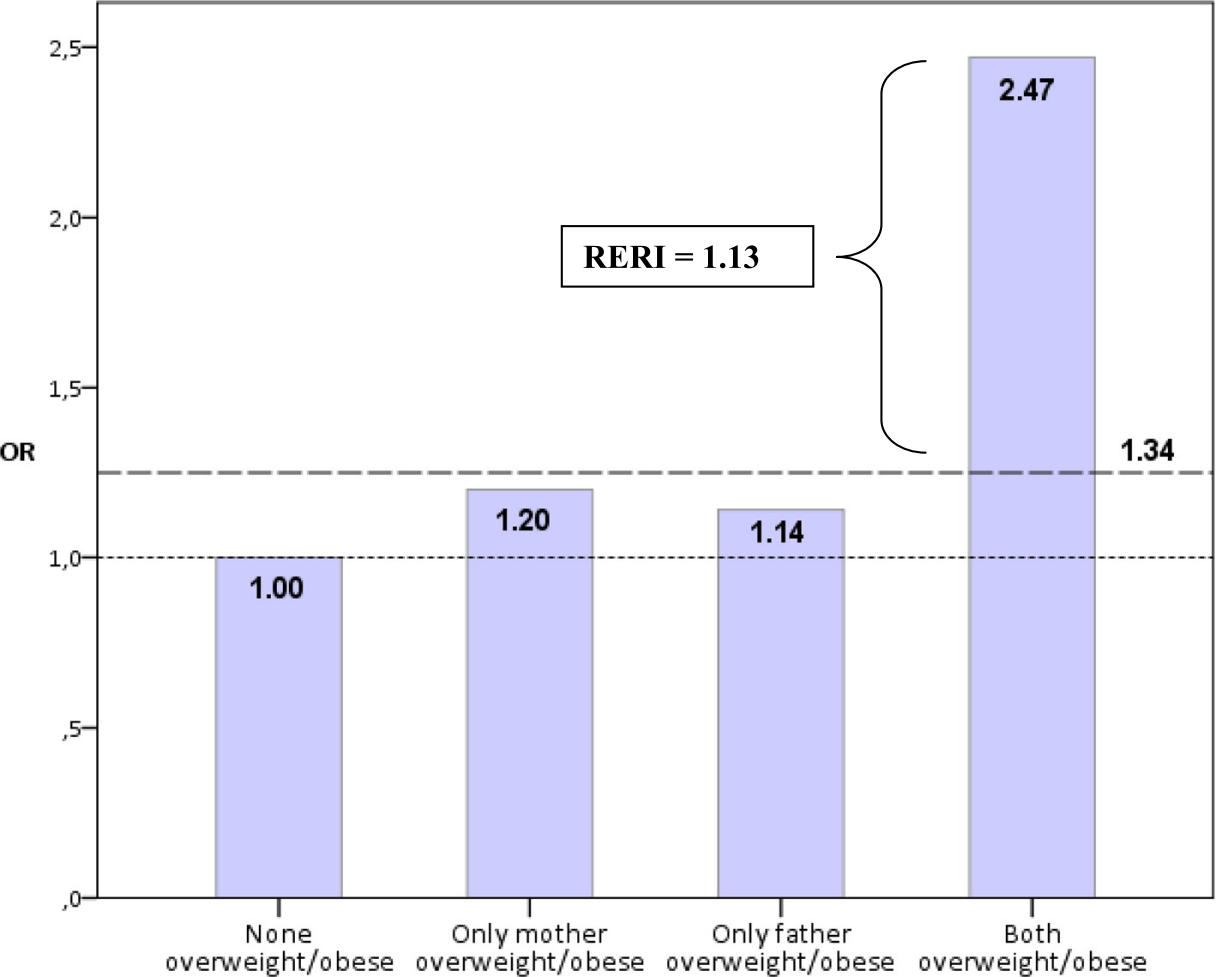
Toddler’s relative odds for a BMI above the WHO 85^th^ percentile when mother and/or father were overweight/obese. RERI illustrates the excess odds when interaction between the mother and father is taken into account

The RERI value was 1.13 in the crude analysis, and this indicates an additive interaction concerning the odds of a child having a BMI above the WHO 85^th^ percentile (Table 4). RERI is here interpreted as the part of the total OR for a child with both parents overweight/obese to have a BMI above a threshold that is due to interaction between the mother and father (Table 4, Fig. 1).

### Associations between toddlers’ BMI and parents’ BMI in relation to socio-demographics

Crude logistic regression analyses of socio-demographic variables in relation to the toddler’s BMI status showed few significant associations. Analyses were performed for education level, employment status, country of origin, family situation, siblings, type of housing, parental age, day care, and child age (in months). In the adjusted analyses, only country of origin remained significant, although with a large confidence interval. Effects concerning parent’s BMI group in relation to children’s BMI status remained (Table 4).

## Discussion

Our main finding was that high BMI values are common already among children aged 16–24 months. A total of 14 % of our sample had a BMI above the WHO 95^th^ percentile and the group corresponding to the WHO 85^th^ percentile made up a total of 33 % of our sample. The probability of a child having a BMI above the WHO 95^th^ percentile was significantly increased if either the mother or the father was overweight/obese. We found a positive synergistic effect between the mother’s and father’s reported overweight/obesity and a child having a BMI above the WHO 85^th^ percentile, i.e. the odds for a child to have a BMI above the WHO 85^th^ percentile BMI was considerably higher if both parents were overweight/obese than if only the mother or father was overweight/obese. No associations were found between the toddlers’ BMI and parental socio-demographics. On the other hand, associations between parental BMI and socio-demographics were found.

The toddlers in our sample had a BMI-status which is considered high. The prevalence of children with high BMI depends both on the reference population and the methodology used for the thresholds [28–30], and major differences appear before the age of 5 years [28]. In light of this, it is difficult to compare our prevalence estimates with results from other countries. There are few data on the BMI status of toddlers younger than the age of 2 years, and we have therefore chosen not to use the terms overweight or obesity for the toddlers in our sample. However, comparing our results with a study of overweight and obesity in children (2.0–9.9 years) from eight European countries [30] shows that our study sample has somewhat higher age-specific prevalence values of BMI status according to the WHO references. The study with European children reports that the prevalence of children with BMI above 1 SDS at the age of 2 years is approximately 26 %. Our study shows that the prevalence of children aged 16–24 months with BMI above the WHO 85^th^ percentile (equal to SDS = 1.036) is 33 %, which is considered high. Another study with Danish preschool children [31] used cut-off values for overweight or obesity according to the international obesity task force group [32]. These cut-off values for overweight in the age group 2.5-3.5 years correspond to values around the WHO 95^th^ percentile. The Danish study showed that approximately 10 % of the children in the age group 2.5-3.5 years had BMI-values above the WHO 95^th^ percentile compared to 14 % in our sample. Recent research shows that fast growth during early childhood was associated with increased risk for overweight later in life, emphasising the importance of early prevention [33]. Childhood obesity and related risk factors have been shown to track into adulthood and to worsen in most individuals [34]. Our findings strengthen recent research showing the importance of early identification and early intervention in children with high BMI [35–37].

Presence of overweight/obesity among mothers and fathers were significantly associated to the probability of their child having a BMI above the WHO 95^th^ percentile. Health promotion activities early in life from a family perspective need to be strengthened by taking into account parental BMI. Our findings reinforce the idea that health-promoting interventions should start already during pregnancy [4]. The evidence regarding the effectiveness of parental-support interventions targeting 2–18 year old children’s health behaviours is weak, although their effectiveness seems to be generally higher in younger compared to older children [38]. Counselling might be effective in changing children’s diet, but concerning body weight group education seems more promising than counselling. In groups with low socioeconomic status group-based methods appear promising [38].

The results in this study thus indicate that the combined effect of the mothers and fathers overweight/obesity is essential for the toddlers’ BMI, as we found a positive synergistic effect between the mother’s and father’s reported overweight/obesity and their child having a BMI above the WHO 85^th^ percentile. This confirm research that shows the importance of considering childhood health and overweight in a family perspective [16] and that there is a risk of a vicious cycle between generations [39].

The rather rapid rise in obesity over the past four decades stresses the complex role of hereditary, socioeconomic, geographic, and environmental factors that influence mechanisms of body weight and weight distribution [18]. We could not find any associations between toddler’s BMI and family’s socio-demographics, but we did find associations between parents’ BMI and parents’ socio-demographics. This shows that even though we cannot see a direct connection between family characteristics and the child’s BMI status in this study, the connection is indirect through the parents. Overall, child health and development is closely related to socioeconomic factors [40, 41]. Gaps between wealthy and poor children are becoming wider in some countries, with increased exposure to health risks combined with less resistance to diseases and health problems in those less privileged [41]. In a recent European study, adverse child health and developmental outcomes were associated with key social factors in the community/neighbourhood as well as in the household [40].

There are few data on the BMI status of toddlers younger than the age of 2 years which makes this study distinctive. Strengths of our study were that child weight and height were measured by experienced child health nurses within the CHC during ordinary health check-ups and that the BMI values were calculated using the WHO Child Growth standards resulting in the exact weight and length at the time of the investigation. In addition, there were very few missing values within the questionnaires. The prevalence of overweight and obesity in our sample of parents was high and, in accordance with previous data [3, 4].

A weakness in the data collection was that parental weight and height were self-reported, and a certain underestimation of weight can be expected [42]. The prevalence of children with high BMI depends on the reference population, and a higher prevalence of overweight/obesity is often observed using the WHO reference values [29, 30], which might overestimate the number of children with high BMI status in this study. Another limitation of the study was that those parents who could not read Swedish were excluded as the questionnaire was only in Swedish, which probably contributed to the lower proportion of immigrants in the study group. Further, the data material in this study consists mainly of categorical variables, and small numbers for some categories means that the power in certain analyses might be low. This makes it hazardous to draw strong conclusions about the non-significant relationships between toddlers’ BMI and families’ socio-demography.

It is difficult to describe the response rate in this sample because research was not the main priority when the data collection started within ordinary health care and because the implementation of the questionnaire was performed gradually. The Umeå SIMSAM Lab (www.org.umu.se/simsam/english/) was used to access data from the whole Swedish population as a comparison to our sample. In our sample, more parents were gainfully employed compared to the rest of Sweden (when the child was 1½ years old), and our sample had a lower proportion of immigrants. Therefore, one can assume that our study parents were more privileged and had higher living standards and therefore would have a somewhat lower BMI compared with the rest of Sweden because overweight/obesity is more common among the most underprivileged groups [3, 4].

## Conclusions

In this population, high BMI values are common already among toddlers. The risk increases if one parent is overweight or obese and increases even more if both parents are affected. Thus our results highlight the importance of considering familial risk factors when examining the child’s health and BMI at ordinary CHC visits already at an early age. The WHO European review of social determinants of health emphasizes the need for action on social determinants of health across the life course, and it recommends that the highest priority be given “to ensure a good start to life for every child” [43]. The Strategic Development Office and its Public Health Unit and the Child Health Care Unit in the Västerbotten County Council of Sweden are now working together towards phasing in psychosocial health surveillance countywide for 3 year olds, together with data on lifestyle and BMI, as well as a group-based parental support program. By placing children’s health and BMI within a life course and family perspective, this continuous data collection will provide a unique resource for further research on childhood health and obesity that will begin to fill in the remaining knowledge gaps [18]. The on-going county-wide implementation of a parental support program targeting health behaviours might likewise be helpful to prevent overweight and obesity, especially for parents with low socioeconomic status [38].
